# GIEHP: A global, AI-powered platform for near real-time ecological intelligence

**DOI:** 10.1016/j.ese.2025.100634

**Published:** 2025-11-19

**Authors:** Dong Xu, Yi-Chen Wang

**Affiliations:** Department of Geography, National University of Singapore, Singapore, 117568, Singapore

The rapid advancement of multi-source remote sensing, cloud computing, and artificial intelligence has transformed the monitoring and assessment of global ecosystems [1]. International initiatives, such as Global Forest Watch [2] and the Group on Earth Observations [3], have demonstrated the importance of global, near-real-time environmental information for large-scale ecological monitoring, providing a better understanding of climate change. Nevertheless, these platforms typically focus on a specific domain, such as forest cover or water resources, and are unable to comprehensively integrate multi-domain and multi-functional ecological indicators within a unified technical framework. We argue that a globally consistent ecological intelligence platform with broader coverage, higher resolution, and integrated multi-domain data will substantially enhance our understanding of global ecological dynamics and climate change.

Here, we introduce the Global Intelligent Ecological Horizon Project (GIEHP), an open, cloud-based platform designed to provide a unified infrastructure for ecological monitoring and data-sharing. GIEHP integrates Earth observation, cloud computing, big data analytics, and geographic artificial intelligence (GeoAI) within an AI-enabled modular architecture [4]. The technical architecture comprises four core modules: multi-source heterogeneous data integration, a model library, a dual computing framework, and an interactive front-end/back-end interface (Fig. 1a). The modular design supports automated workflows, cross-domain analysis, and scalable model deployment. The platform enables the near-real-time computation of more than 300 ecological indicators across terrestrial, aquatic, atmospheric, and urban systems, with spatial resolutions ranging from 4 m to 1 km, spanning from 2000 to the present. These indicators are accessible through both a web portal (https://www.giehp.cn or https://www.giehp.com) and an application programming interface. All GIEHP datasets are made available free of charge under the Creative Commons Attribution–NonCommercial 4.0 International (CC BY-NC 4.0) license, allowing non-commercial use, redistribution, and adaptation with appropriate attribution.

**Fig. 1:**
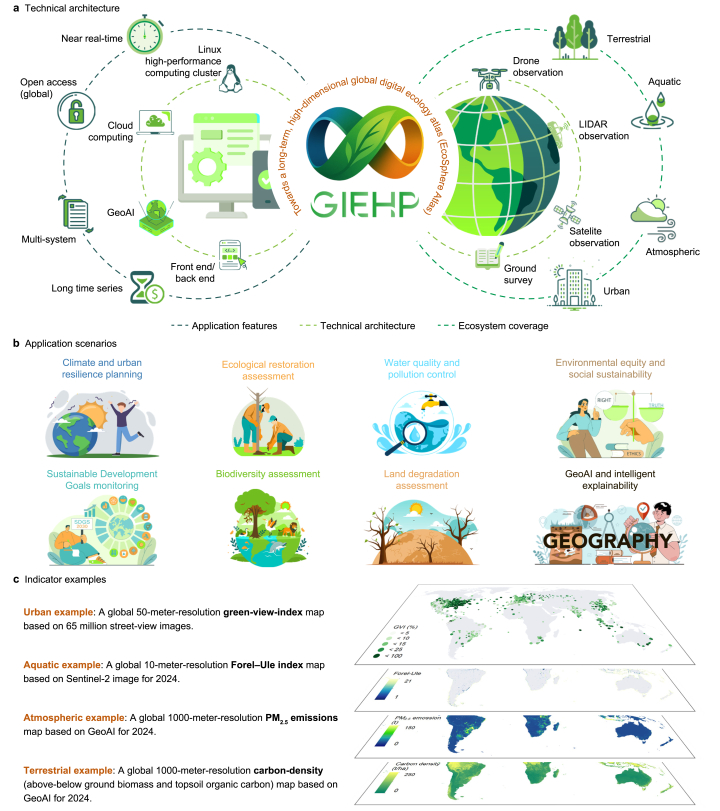
Technical architecture, ecosystem coverage, application features, and example data of the Global Intelligent Ecological Horizon Project (GIEHP). a, The technical architecture, ecosystem coverage, and application features of GIEHP. **b**, Application scenarios of GIEHP. **c**, Example data products for four major ecosystem types, including global green-view-index (GVI) maps derived from street-view imagery [10] (urban), global Forel–Ule water-color index maps [11] (aquatic), global anthropogenic PM_2.5_ emissions maps (atmospheric), and global terrestrial carbon-density maps (terrestrial). Blank areas in panel **b** represent non-applicable regions rather than missing data. The urban example is computed only for urban extents, while the aquatic example (water-color index) is limited to inland and nearshore water bodies.

GIEHP supports scientific research and policy-oriented applications across multiple ecological and socio-environmental domains. The platform can be applied to diverse areas, including climate and urban resilience planning, ecological restoration assessment, and other fields (Fig. 1b). Collectively, this architecture establishes the computational and data backbone [5] for developing a next-generation intelligent digital earth atlas (EcoSphere Atlas), which represents the central objective of GIEHP. The EcoSphere Atlas is designed to integrate long-term temporal consistency with high-dimensional feature representation. It conceptualizes the Earth as a high-dimensional raster data cube that unifies natural and human processes within a consistent spatiotemporal framework. Through this integration, the system substantially enhances the interpretability of global surface dynamics and the underlying driving mechanisms of climate change [6,7], thereby providing a robust data foundation for cross-scale prediction, integrated assessment, and scenario simulation. The four representative ecological indicators of the four major ecosystems presented (Fig. 1c) are intended to demonstrate the diversity of ecological subsystems and the multidimensional analytical capability of GIEHP [8]. More indicator examples can be found in Supplementary Fig. S1–S15.

GIEHP addresses the pressing demand for an integrated, high-resolution, and openly accessible platform for global ecological monitoring. It offers comprehensive coverage, regular updates, and customizable analytical scales. Future development of GIEHP will expand its data coverage to the marine domain by incorporating oceanic variables (e.g., sea surface temperature, chlorophyll-*a*, net primary production, and CO_2_ fluxes), thereby building a fully integrated land–ocean–atmosphere ecological intelligence framework. We encourage researchers from diverse disciplines to actively engage in the development and maintenance of GIEHP. Through collaborative efforts to strengthen the system architecture, broaden the indicator set, and refine algorithmic performance [9], the platform can continue to evolve and advance as an open, shared global resource.

## CRediT authorship contribution statement

**Dong Xu:** Writing – review & editing, Writing – original draft, Visualization, Validation, Supervision, Software, Resources, Project administration, Methodology, Investigation, Funding acquisition, Formal analysis, Data curation, Conceptualization. **Yi-Chen Wang:** Writing – review & editing, Supervision, Project administration, Investigation, Funding acquisition, Formal analysis, Data curation, Conceptualization.

## Declaration of competing interest

The authors declare that they have no known competing financial interests or personal relationships that could have appeared to influence the work reported in this paper.
