# Supplementary material for: GIEHP: A global, AI-powered platform for near real-time ecological intelligence
Source: Environ Sci Ecotechnol. 2025 Nov 19;28:100634. doi: 10.1016/j.ese.2025.100634 (PMC12681839; doi:10.1016/j.ese.2025.100634)
Supplement: Multimedia component 1 [file mmc1.doc]

## **Supplementary information for**

## **GIEHP: A Global, AI-powered platform for near real-time ecological intelligence**

Dong Xu^*^, Yi-Chen Wang

Department of Geography, National University of Singapore, Singapore 117568, Singapore.

*Corresponding author: Dong Xu (Email: xu.dong@u.nus.edu)

ORCID: 0009-0002-8238-2665 (Dong Xu), 0000-0002-3034-7377 (Yi-Chen Wang)

**This Supplementary Information file includes:**

Number of pages: 16

Figures: S1–S17

The indicators provided by GIEHP include both foundational technical datasets and advanced application metrics covering four major ecological subsystems: terrestrial, aquatic, atmospheric, and urban. We have added representative examples to illustrate their scope and application.

- ***Terrestrial ecosystem***


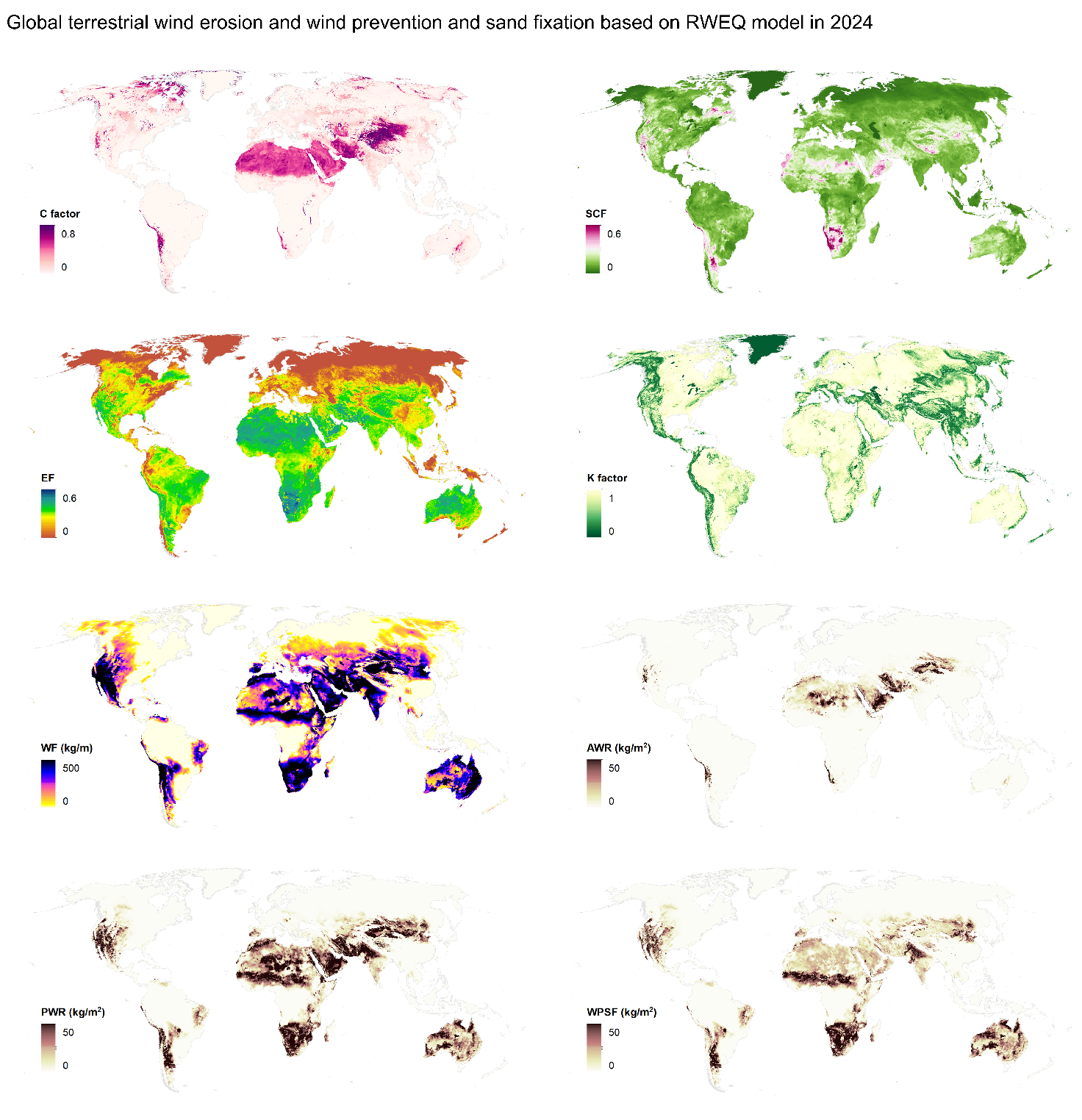


**Fig. S1 | Global terrestrial 1 km resolution wind erosion and wind prevention and sand fixation based on RWEQ model in 2024.** C represents the vegetation factor; SCF denotes the soil crust factor; EF refers to the soil erodibility factor; K indicates the surface roughness factor; WF is the climatic factor (kg/m); AWR represents the actual wind erosion amount (kg/m^2^); PWR denotes the potential wind erosion amount (kg/m^2^); and WPSF refers to the wind prevention and sand fixation amount (kg/m^2^). For more information about the RWEQ model, please refer to below paper ^[1]^.

1. Fryrear, D. W., Bilbro, J. D., Saleh, A. R. W. E. Q., Schomberg, H., Stout, J. E., & Zobeck, T. M. (2000). RWEQ: Improved wind erosion technology. *Journal of soil and water conservation*, *55*(2), 183-189.


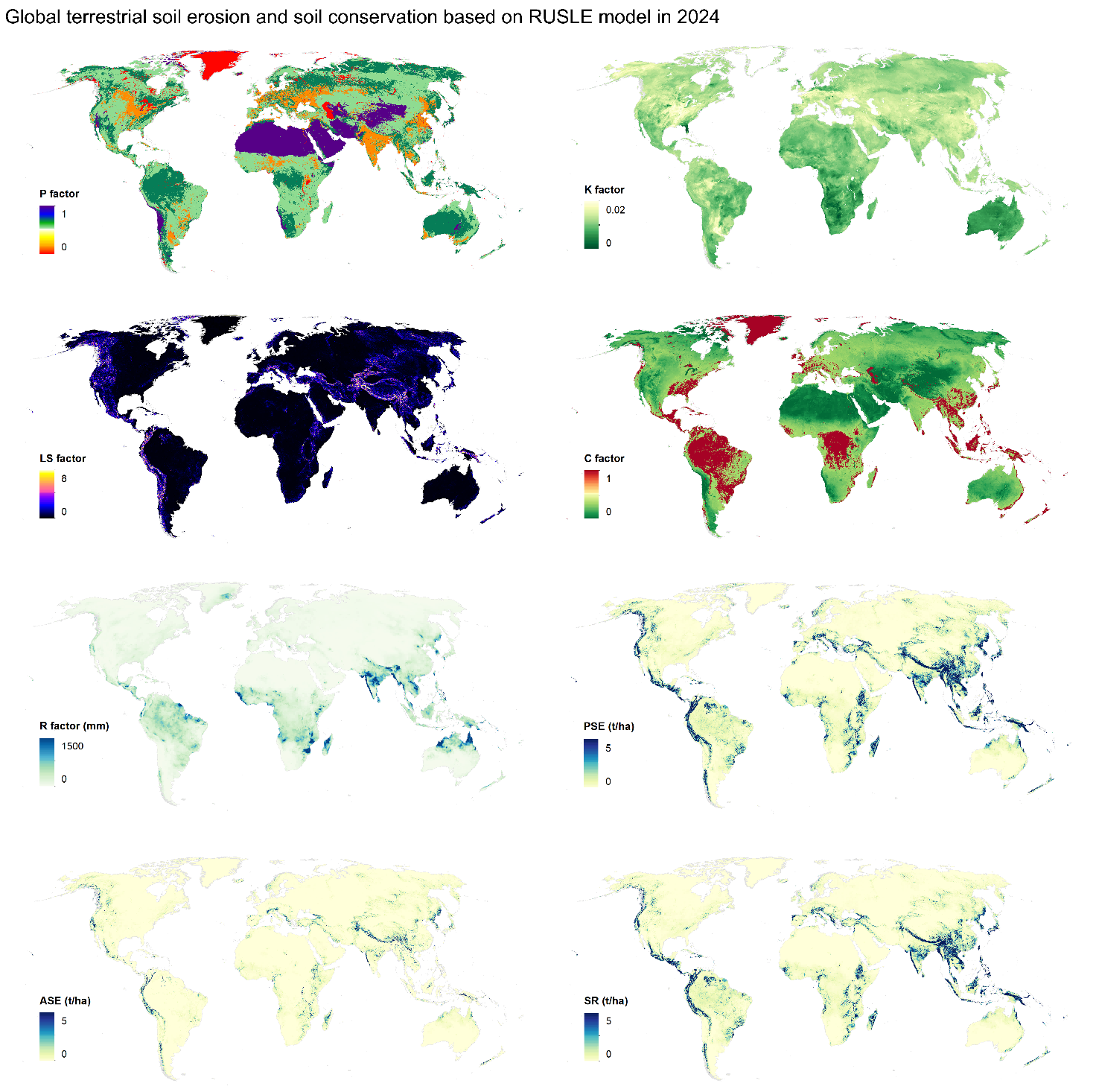


**Fig. S2 | Global terrestrial 1 km resolution soil erosion and soil conservation based on RUSLE model in 2024.** P represents the conservation practice factor; K denotes the soil erodibility factor; LS refers to the slope length and steepness factor; C indicates the vegetation cover factor; R is the rainfall erosivity factor (MJ·mm·hm^-2^·h^-1^·a^-1^); ASE represents the actual soil erosion amount (t/ha); PSE denotes the potential soil erosion amount (t/ha); and SR refers to the soil retention amount (t/ha). For more information about the RUSLE model, please refer to below paper ^[2]^.

1. Renard, K. G., & Ferreira, V. A. (1993). RUSLE model description and database sensitivity. *Journal of environmental quality, 22*(3), 458-466.


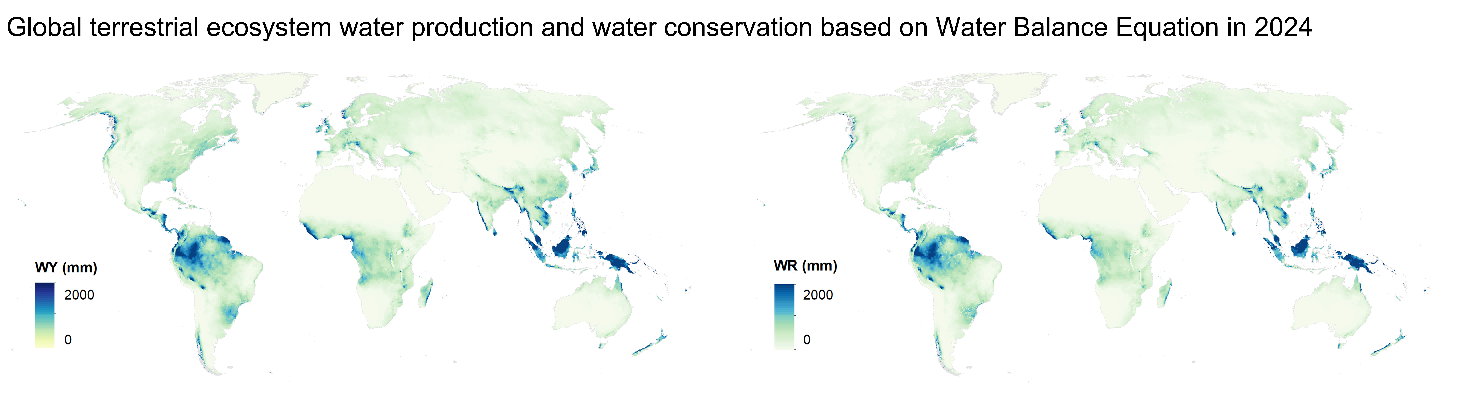


**Fig. S3 | Global terrestrial 1 km resolution water production and water consevation based on Water Balance Equation in 2024.** WY (mm) represents the annual water yield, and WR (mm) denotes the annual water retention (or water conservation). For more information about the Water Balance Equation, please refer to below papers ^[3]-[4]^.

1. Redhead, J. W., Stratford, C., Sharps, K., Jones, L., Ziv, G., Clarke, D., ... & Bullock, J. M. (2016). Empirical validation of the InVEST water yield ecosystem service model at a national scale. *Science of the Total Environment, 569*, 1418-1426.
2. Yang, D., Liu, W., Tang, L., Chen, L., Li, X., & Xu, X. (2019). Estimation of water provision service for monsoon catchments of South China: Applicability of the InVEST model. *Landscape and Urban Planning, 182*, 133-143.


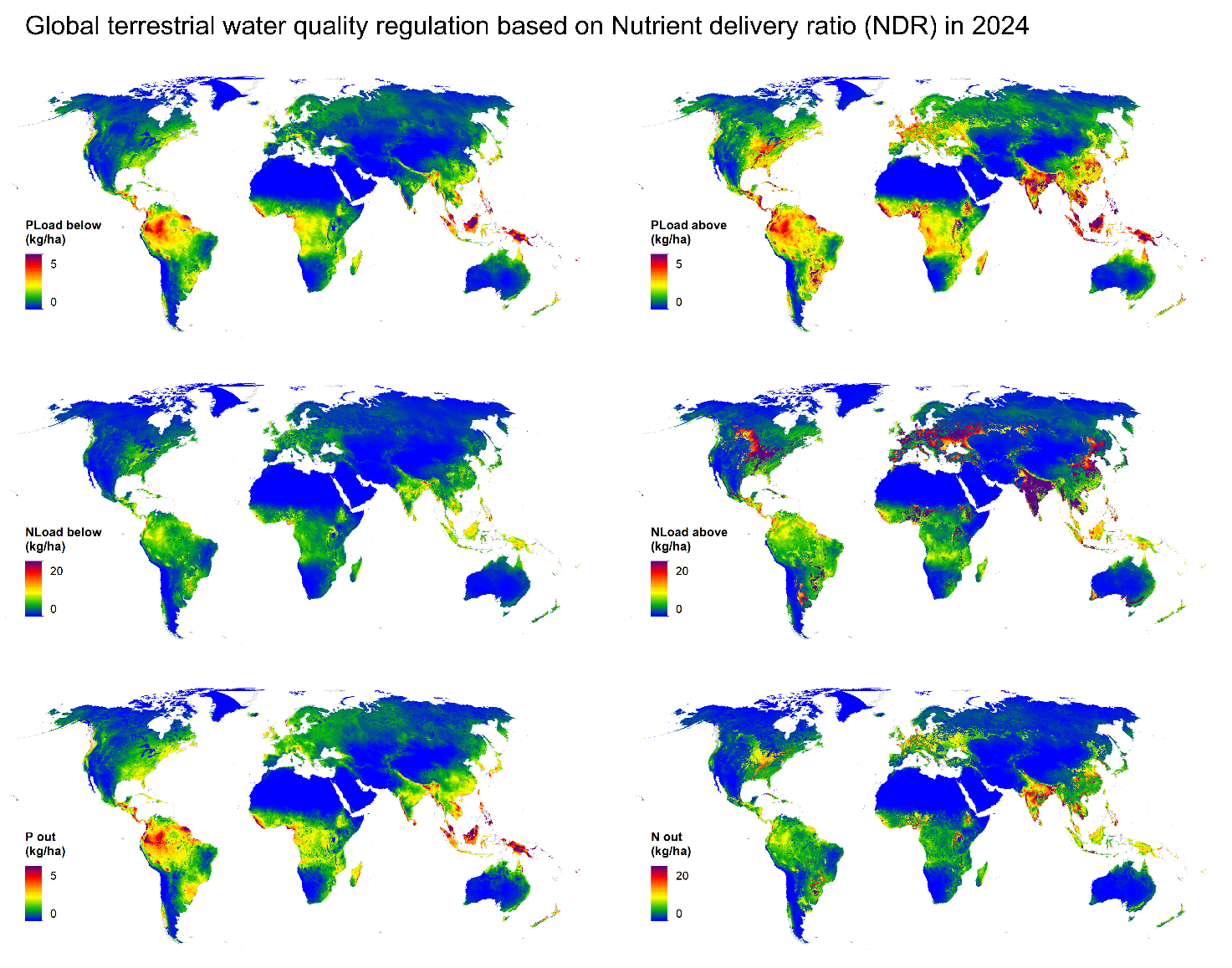


**Fig. S4 | Global terrestrial 1 km resolution water quality regulation based on Nutrient delivery ratio (NDR) in 2024.** PLoad below (kg/ha) represents the subsurface phosphorus load; PLoad above (kg/ha) denotes the surface phosphorus load; NLoad below (kg/ha) refers to the subsurface nitrogen load; NLoad above (kg/ha) indicates the surface nitrogen load; P out (kg/ha) represents the phosphorus output); and N out (kg/ha) denotes the nitrogen output. For more information about the NDR, please refer to below papers ^[5]-[6]^.

1. Redhead, J. W., May, L., Oliver, T. H., Hamel, P., Sharp, R., & Bullock, J. M. (2018). National scale evaluation of the InVEST nutrient retention model in the United Kingdom. *Science of the Total Environment, 610*, 666-677.
2. Valladares-Castellanos, M., de Jesús Crespo, R., Xu, Y. J., & Douthat, T. H. (2024). A framework for validating watershed ecosystem service models in the United States using long-term water quality data: Applications with the InVEST Nutrient Delivery (NDR) model in Puerto Rico. *Science of the Total Environment, 949,* 175111.


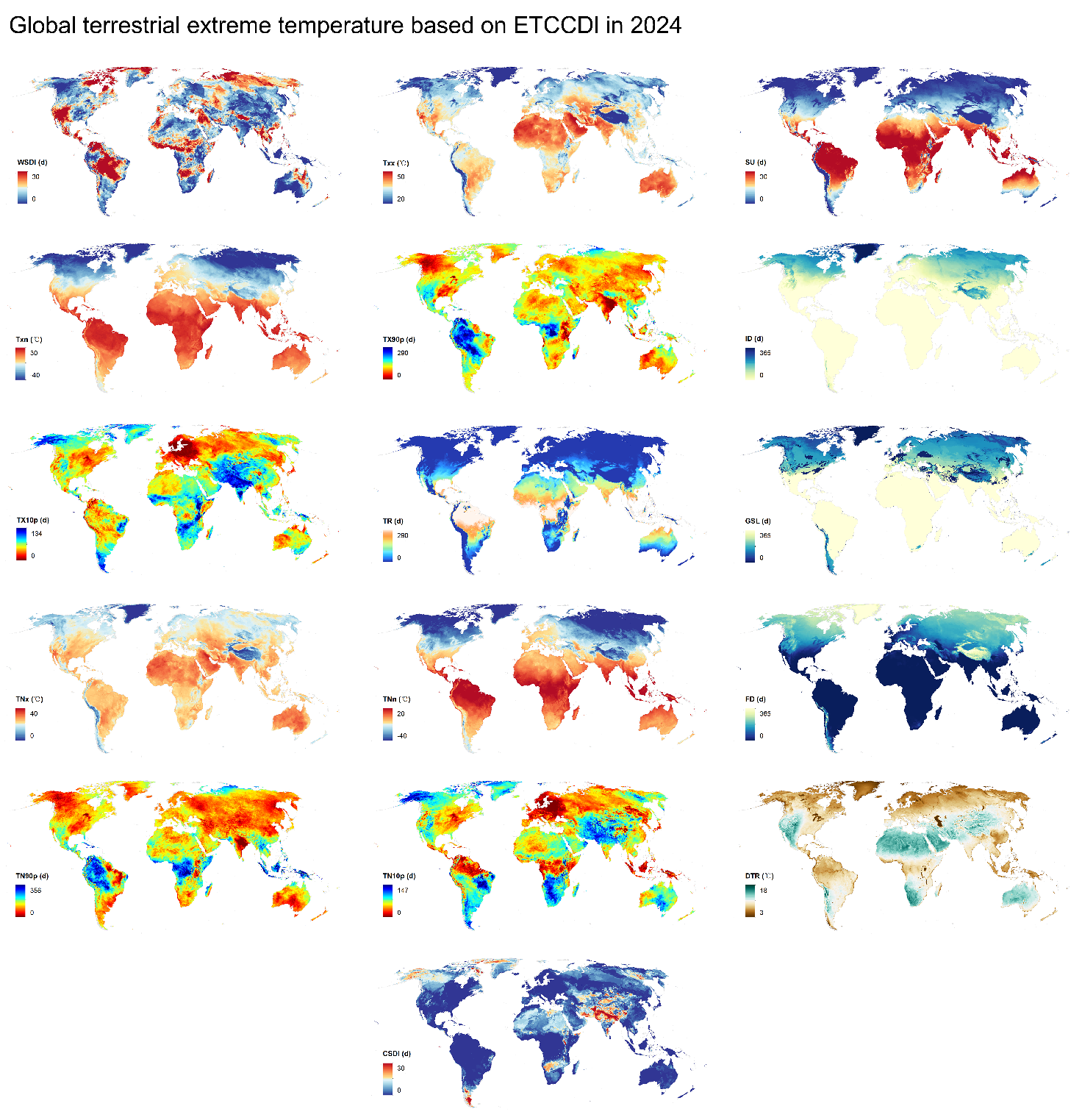


**Fig. S5 | Global terrestrial extreme temperature based on ETCCDl in 2024.** For more information about the ETCCDI, please refer to below papers ^[7]-[8]^.


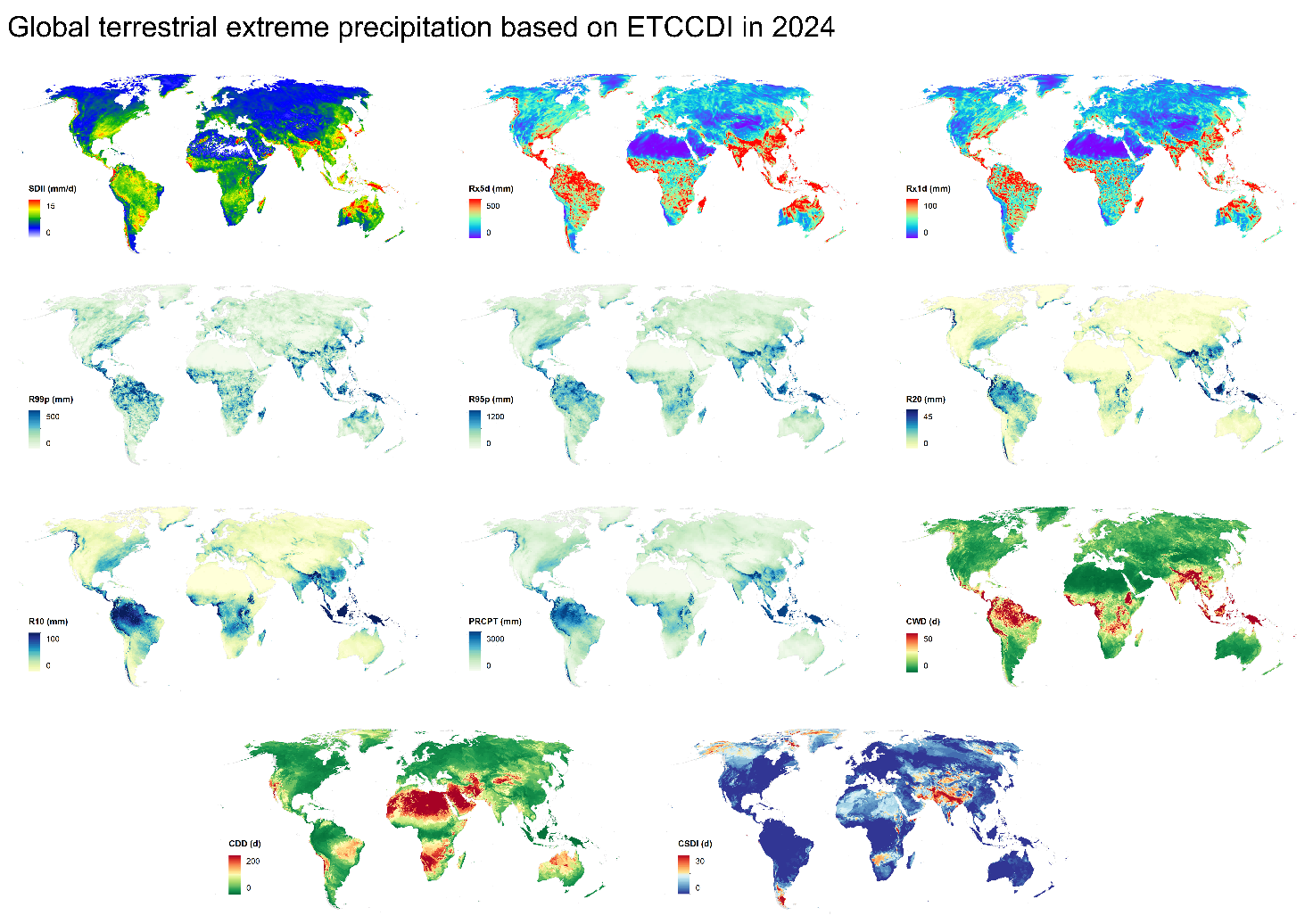


**Fig. S6 | Global terrestrial extreme precipitation based on ETCCDl in 2024.** For more information about the ETCCDI, please refer to below papers ^[7]-[8]^.

1. Hong, Y. I. N., & Ying, S. U. N. (2018). Characteristics of extreme temperature and precipitation in China in 2017 based on ETCCDI indices. *Advances in Climate Change Research, 9*(4), 218-226.
2. Sun, C., Zhu, L., Liu, Y., Hao, Z., & Zhang, J. (2021). Changes in the drought condition over northern East Asia and the connections with extreme temperature and precipitation indices. *Global and Planetary Change, 207*, 103645.


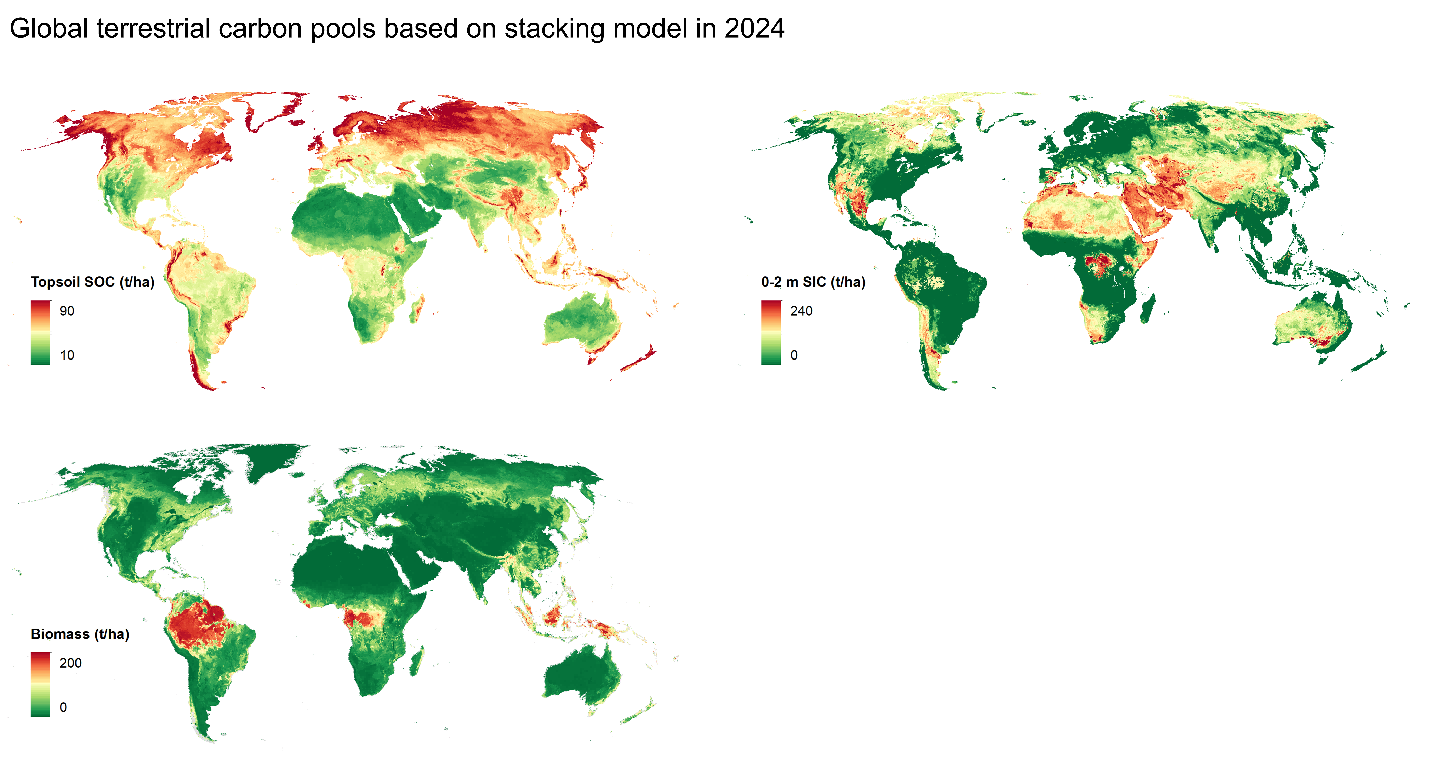


**Fig. S7 | Global terrestrial 1 km resolution carbon pools based on stacking model in 2024.** Since the article associated with these datasets are currently under review, the technical details of this part cannot be disclosed at this stage. Several related publications are provided below for reference ^[9]-[10]^.

1. Mo, L., Crowther, T. W., Maynard, D. S., Van den Hoogen, J., Ma, H., Bialic-Murphy, L., ... & Ontikov, P. (2024). The global distribution and drivers of wood density and their impact on forest carbon stocks. *Nature Ecology & Evolution, 8*(12), 2195-2212.
2. Mo, L., Zohner, C. M., Reich, P. B., Liang, J., De Miguel, S., Nabuurs, G. J., ... & Ortiz-Malavasi, E. (2023). Integrated global assessment of the natural forest carbon potential. *Nature, 624*(7990), 92-101.


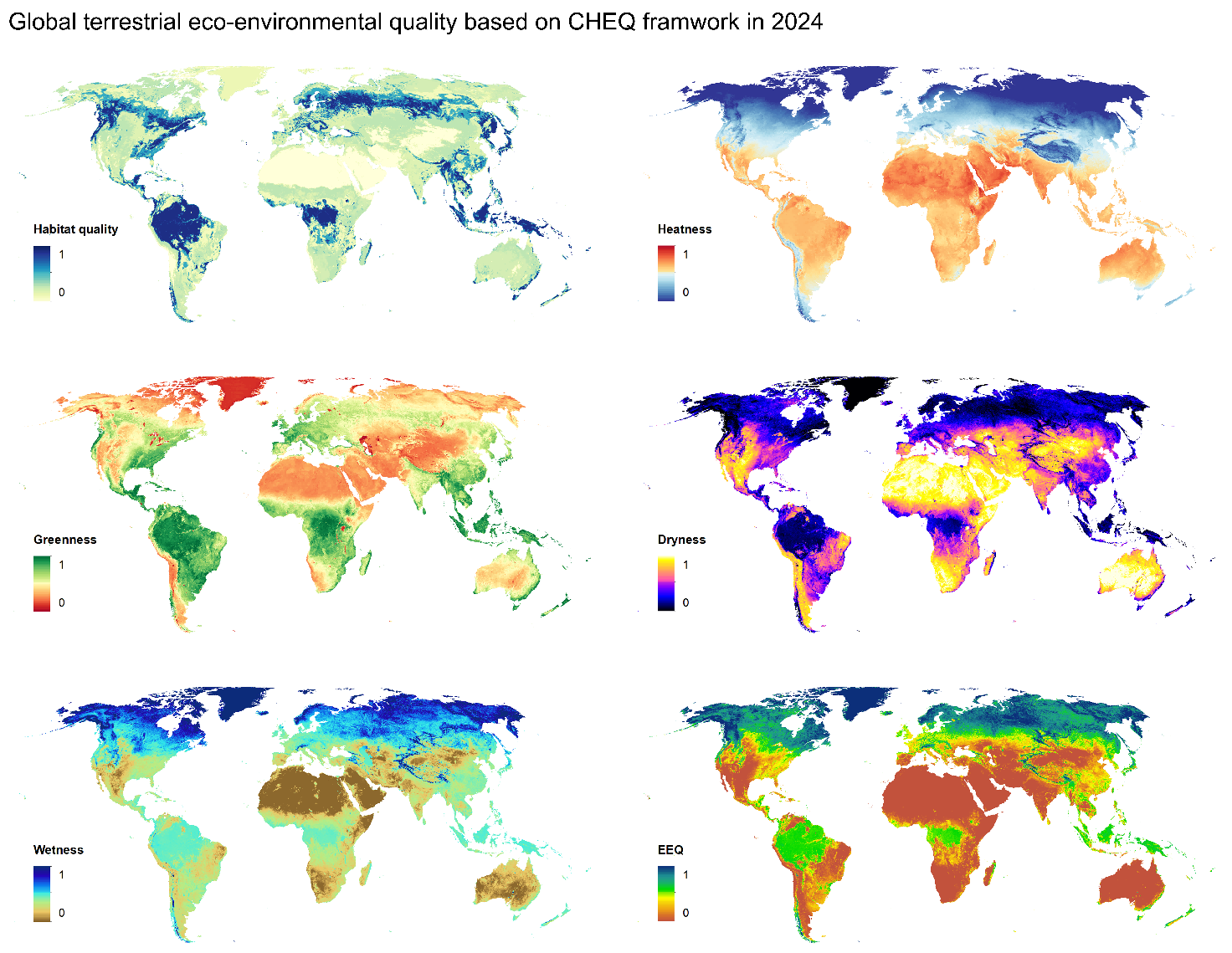


**Fig. S8 | Global terrestrial 1 km resolution eco-environmental quality based on CHEQ framwork in 2024.** For more information about the CHEQ, please refer to below paper ^[11]^.

1. Xu, D., Yang, F., Yu, L., Zhou, Y., Li, H., Ma, J., ... & Cheng, J. (2021). Quantization of the coupling mechanism between eco-environmental quality and urbanization from multisource remote sensing data. *Journal of Cleaner Production, 321*, 128948.


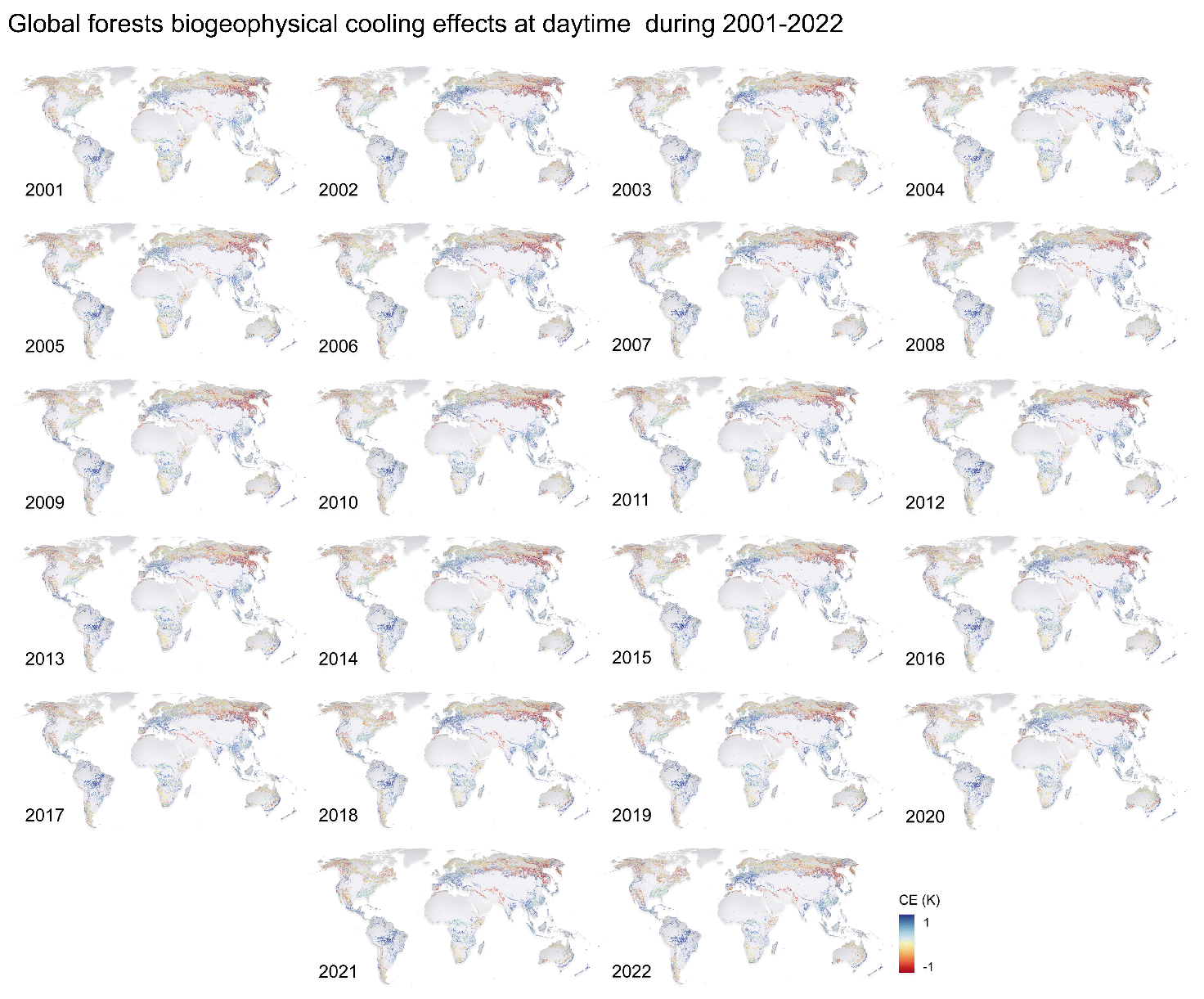


**Fig. S9 | Global 1 km resolution forests biogeophysical cooling effects at daytime during 2001-2022.** Since the article associated with these datasets are currently under review, the technical details of this part cannot be disclosed at this stage. Several related publications are provided below for reference ^[12]-[13]^.

1. Zeng, Z., Piao, S., Li, L. Z., Zhou, L., Ciais, P., Wang, T., ... & Wang, Y. (2017). Climate mitigation from vegetation biophysical feedbacks during the past three decades. *Nature Climate Change, 7*(6), 432-436.
2. Bright, R. M., Davin, E., O’Halloran, T., Pongratz, J., Zhao, K., & Cescatti, A. (2017). Local temperature response to land cover and management change driven by non-radiative processes. *Nature Climate Change, 7*(4), 296-302.

- ***Aquatic ecosystem***


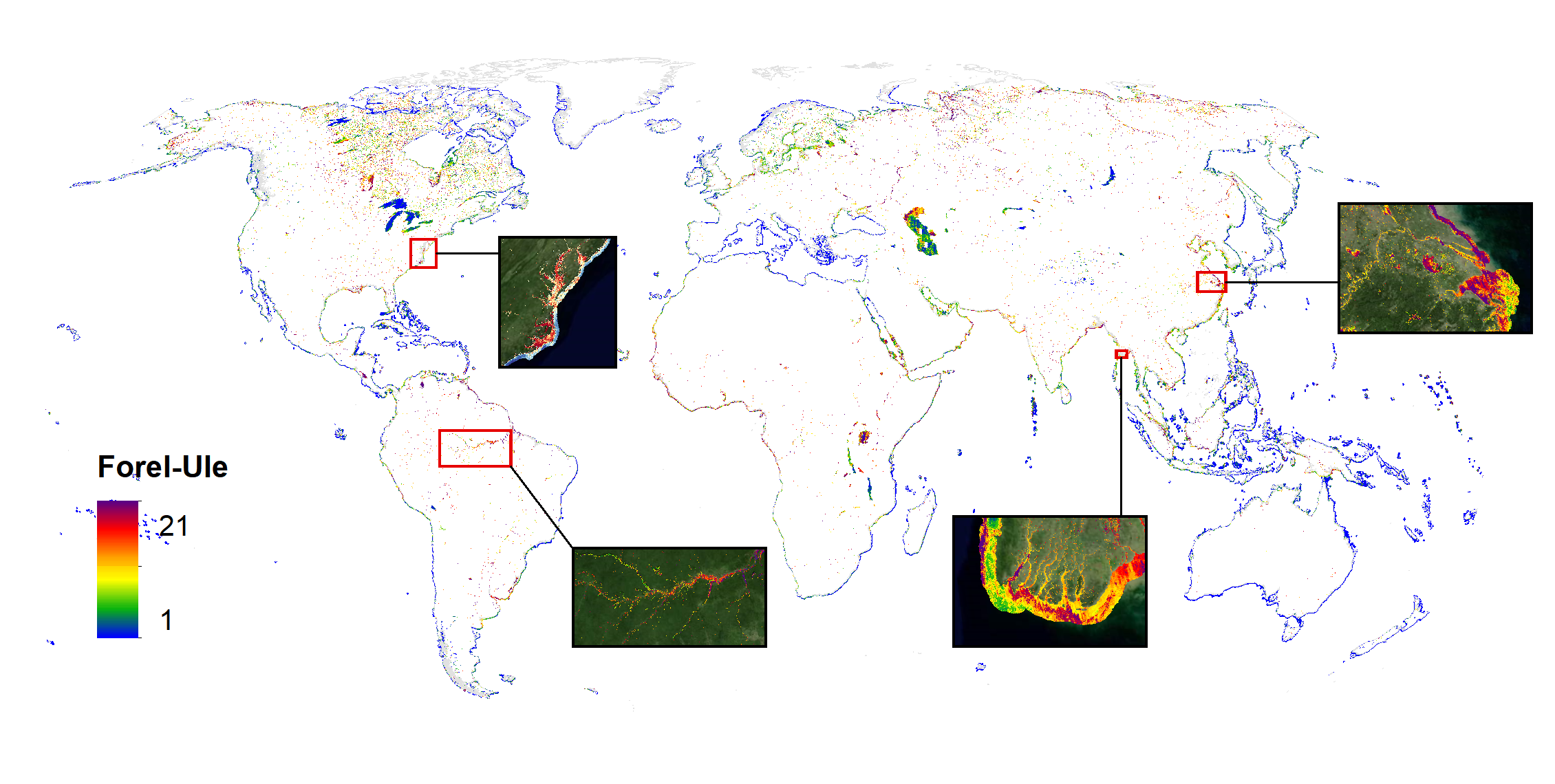


**Fig. S10 | Global 30 m resolution inland and coastal water color based on Forel-Ule index in 2024.** For more information about the Forel-Ule, please refer to below paper ^[14]-[15]^.

1. Shen, X., Ke, C. Q., Duan, Z., Cai, Y., Li, H., & Xiao, Y. (2025). Satellite observations reveal widespread color variations in global lakes since the 1980s. *Water Resources Research, 61*(1).
2. Xia, K., Wu, T., Li, X., & Wang, S. (2024). A new method for accurate inversion of Forel-Ule index using MODIS images-revealing the water color evolution in China's large lakes and reservoirs over the past two decades. *Water Research, 255*, 121560.

- ***Atmospheric ecosystem***


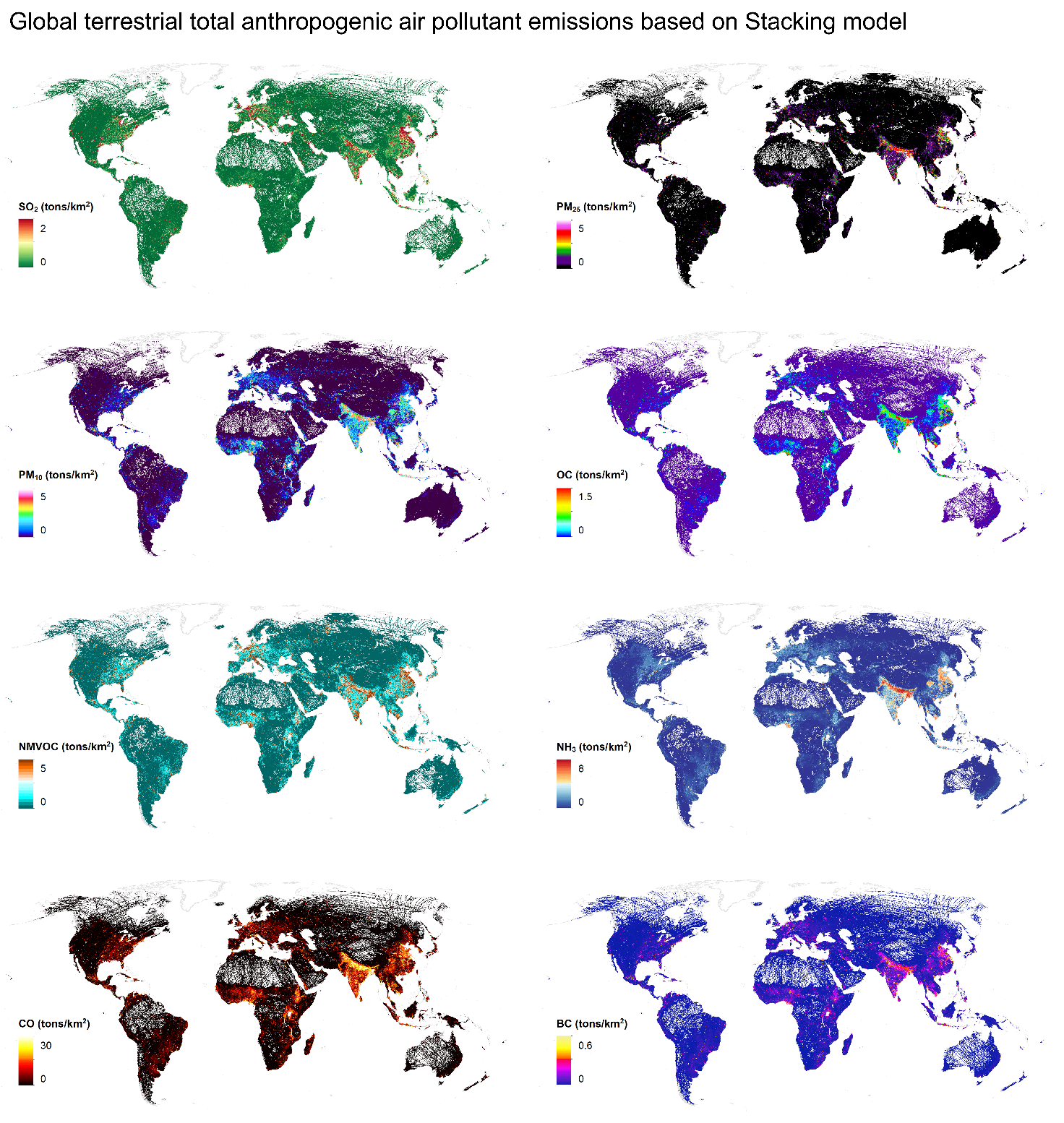


**Fig. S11 | Global 1 km resolution terrestrial total anthropogenic air pollutant emissions based on Stacking model and EDGAR v8.1 in 2022.** EDGAR v8.1 provides a global dataset of anthropogenic greenhouse gas and air pollutant emissions from all sectors at a 10 km spatial resolution for the period 1970-2022. For more information about EDGAR v8.1, please refer to below link and paper ^[16]-[17]^.


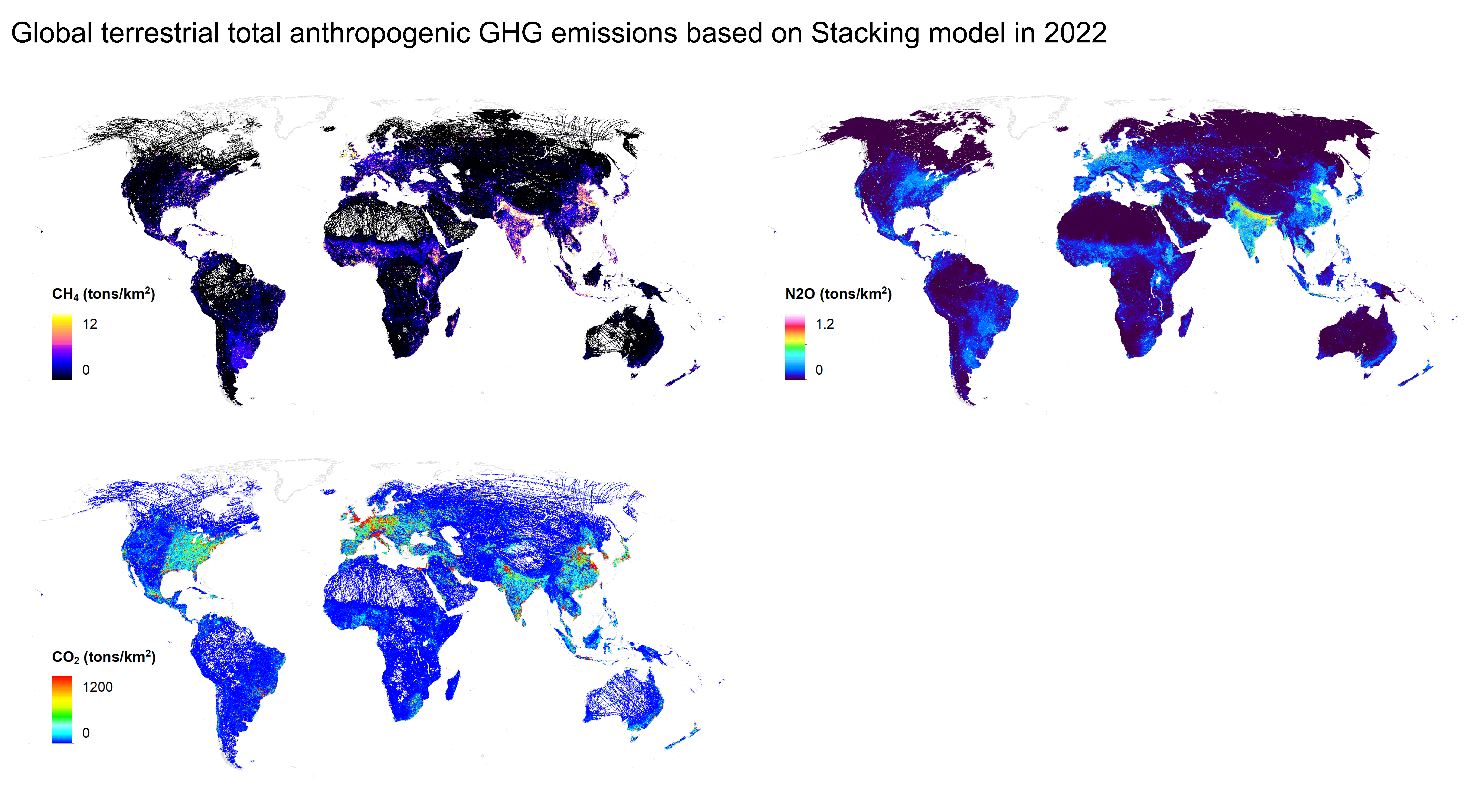


**Fig. S12 | Global 1 km resolution terrestrial total anthropogenic GHG emissions based on Stacking model and EDGAR v8.1 in 2022.** EDGAR v8.1 provides a global dataset of anthropogenic greenhouse gas and air pollutant emissions from all sectors at a 10 km spatial resolution for the period 1970-2022. For more information about EDGAR v8.1, please refer to below link and paper ^[16]-[17]^.

1. <https://edgar.jrc.ec.europa.eu/dataset_ap81>
2. Crippa, M., Guizzardi, D., Pagani, F., Schiavina, M., Melchiorri, M., Pisoni, E., ... & Coheur, P. (2024). Insights into the spatial distribution of global, national, and subnational greenhouse gas emissions in the Emissions Database for Global Atmospheric Research (EDGAR v8. 0). *Earth System Science Data, 16*(6), 2811-2830.

- ***Urban ecosystem***

The indicators of urban ecosystems are derived from 1.05 billion global street-view images using deep learning techniques. The dataset encompasses **6** urban perception dimensions and **150** urban elements (Figs. S13-14). Specifically, the urban perception indicators, including Beautiful, Boring, Safe, Wealthy, Depressing, and Lively, were generated based on the MIT Place Pulse 2.0 dataset using the VGG-16 model. The urban elements, including vegetation, buildings, roads, and sky, were extracted from the ADE20K dataset using a Fully Convolutional Network (FCN) model, resulting in 150 semantic categories. The maps below illustrate the global spatial distribution of urban beauty perception and vegetation coverage at a spatial resolution of 50 m. For more information about Ade20k, please refer to below paper ^[18]^. For more information about MIT Place Pulse 2.0, please refer to below paper ^[19]^.

It is worth noting that a recent study has discussed the ethical and cultural implications of using perception labels such as ‘boring’ or ‘depressing’ in the MIT Place Pulse 2.0 dataset. Rui and Cai ^[20]^ highlighted potential biases in these terms and suggested the need for contextual interpretation when applying them in urban perception research. In this study, these indicators are employed solely as normalized quantitative representations of human perceptual tendencies, rather than evaluative judgments.


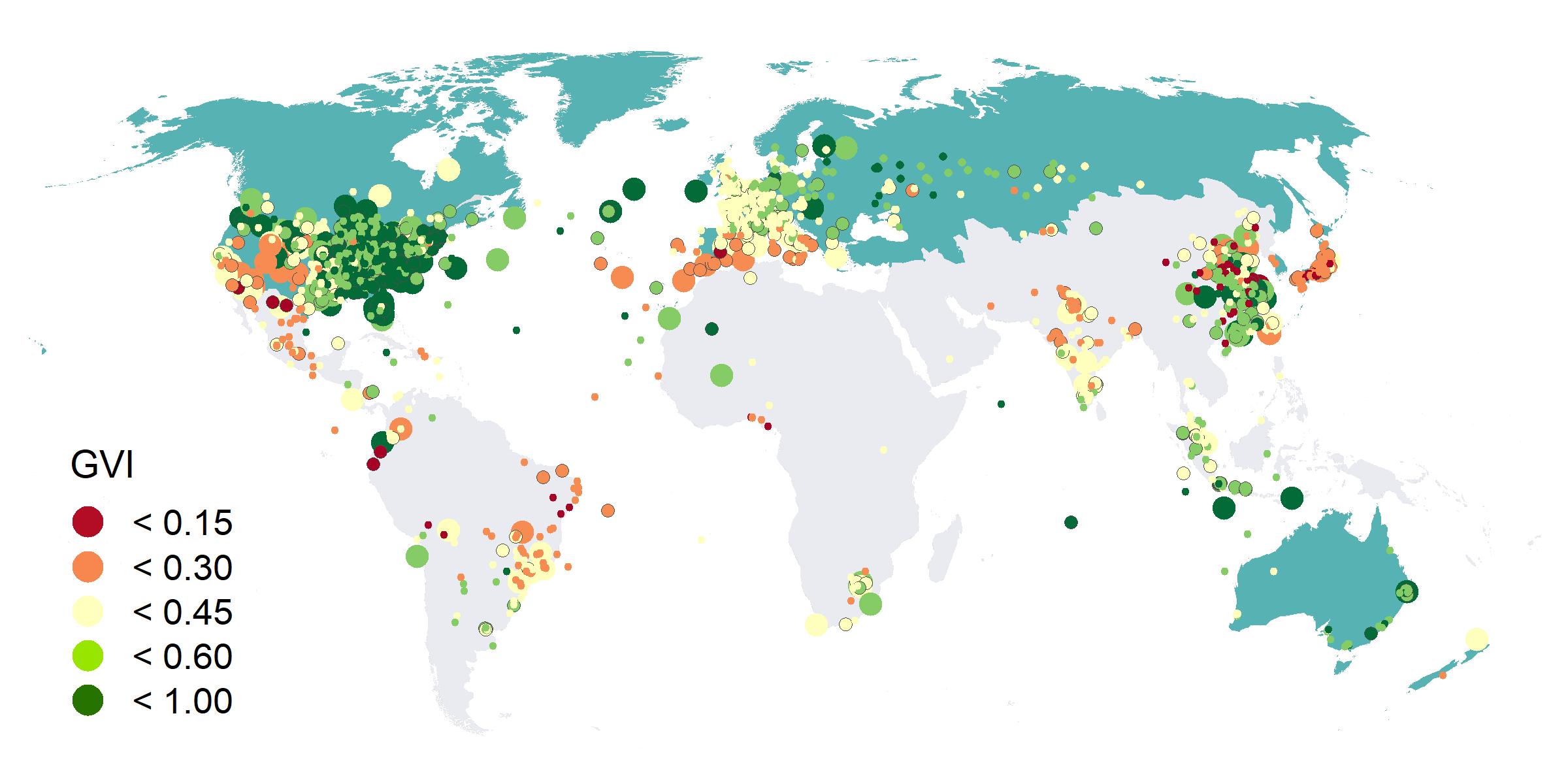


**Fig. S13 | Global urban-scale street view green view index (GVI) distribution map.**


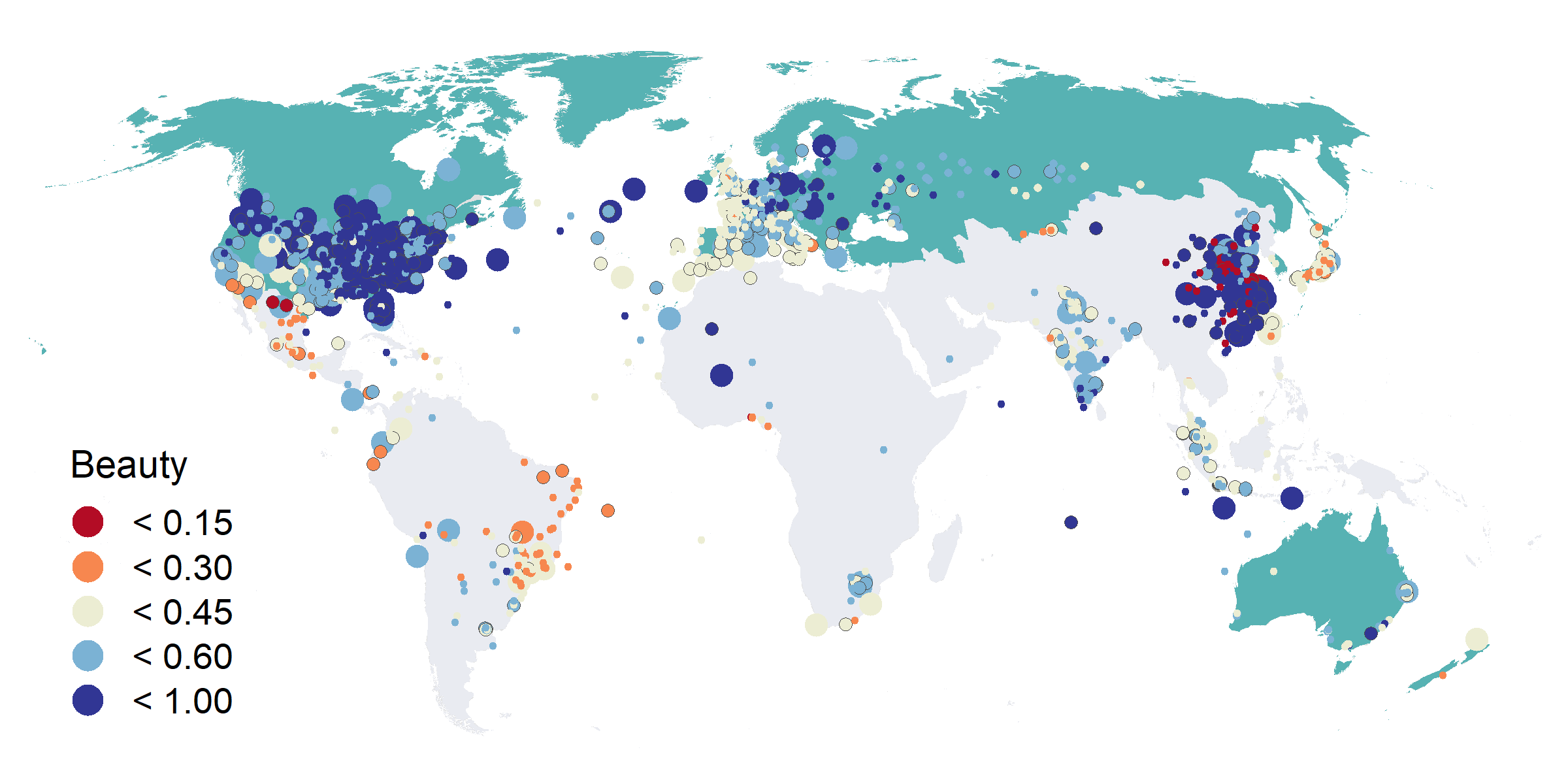


**Fig. S14 | Global urban-scale street view “Beauty” emotion distribution map.**

1. Zhou, B., Zhao, H., Puig, X., Fidler, S., Barriuso, A., & Torralba, A. (2017). Scene parsing through ade20k dataset. In Proceedings of the IEEE conference on computer vision and pattern recognition (pp. 633-641).
2. Dubey, A., Naik, N., Parikh, D., Raskar, R., & Hidalgo, C. A. (2016, September). Deep learning the city: Quantifying urban perception at a global scale. In European conference on computer vision (pp. 196-212). Cham: Springer International Publishing.
3. Rui, J., & Cai, C. (2025). Plausible or misleading? Evaluating the adaption of the place pulse 2.0 dataset for predicting subjective perception in Chinese urban landscapes. Habitat International, 157, 103333.

The apparent difference between Figs. S15-16 and the online map arises from the visualization method rather than any data discrepancy. The figure in the manuscript uses a grid-based static visualization suitable for print and academic presentation, while the live platform adopts a particle-based rendering approach designed for smoother interactivity and dynamic effects. On the website, users can adjust the particle size through the control panel to explore spatial density variations and visualization granularity. The following two figures show the effects of scale factors 1 and 2 respectively.


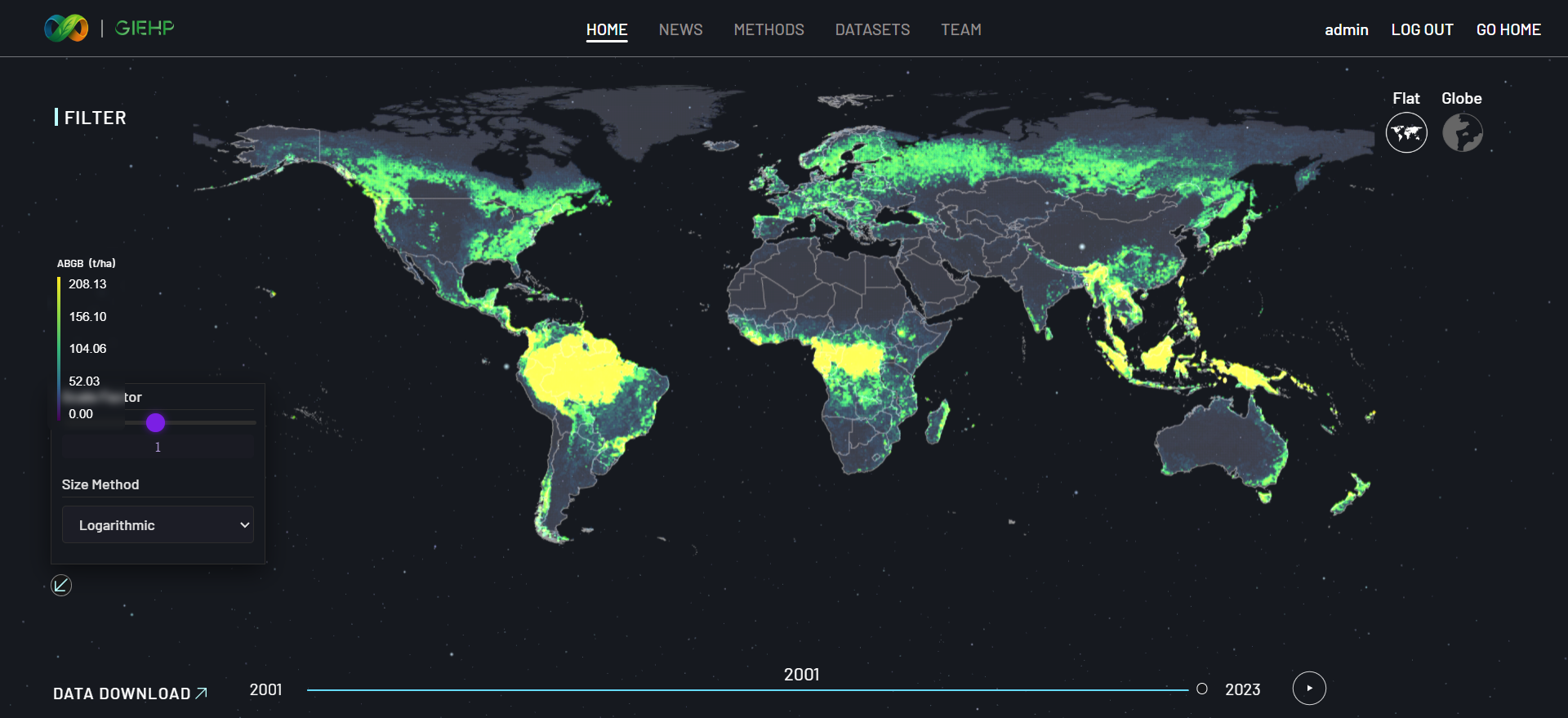


**Fig. S15 | Global biomass spatial pattern when the scaling factor is 1.**


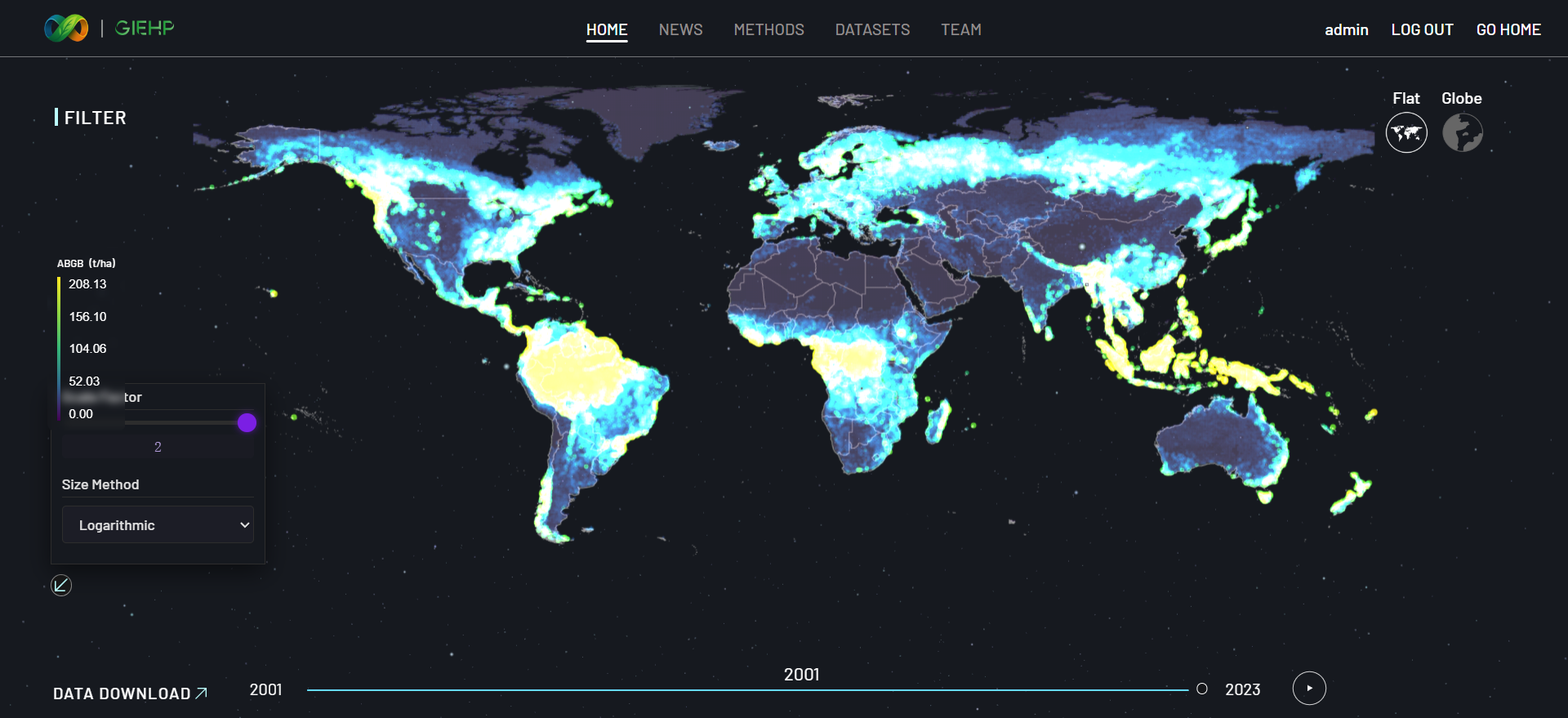


**Fig. S16 | Global biomass spatial pattern when the scaling factor is 3.**

To further demonstrate the near real-time capability of the GIEHP system, we have included an example derived from Landsat 8 and Landsat 9 imagery showing the monthly Forel-Ule water color index for a selected region near Mobile, Alabama, USA during 2025 (Fig. S17). As illustrated in the figure, the water color of this region exhibits a clear transition from turbid conditions in January to much clearer water by October, reflecting the system’s ability to capture fine-scale temporal dynamics in aquatic ecosystems. This example highlights how GIEHP can automatically retrieve and process newly released satellite data to generate near real-time environmental indicators at sub-annual timescales.


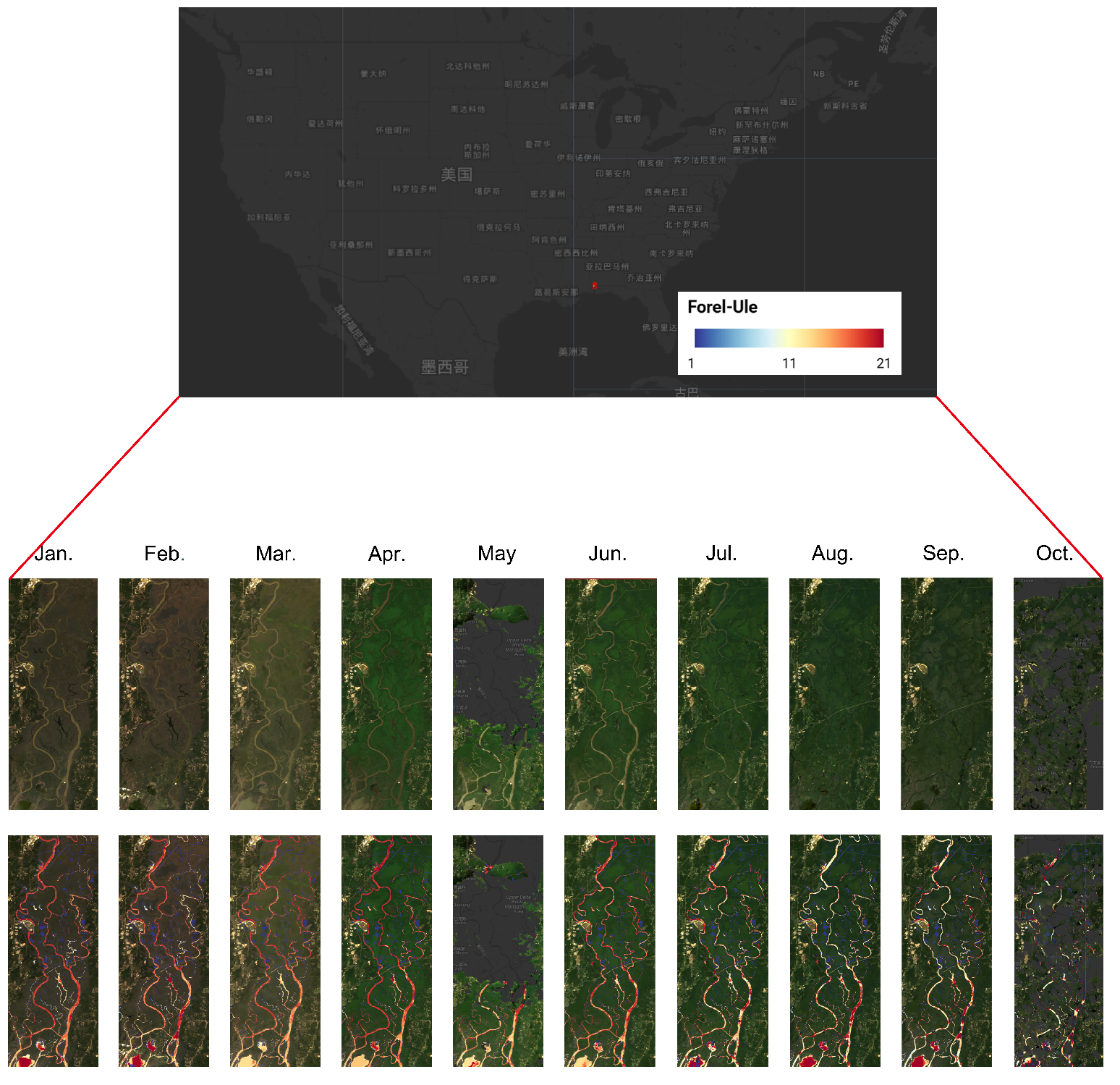


**Fig. S17 | Spatial distribution of the Forel-Ule water color index in the Mobile area of the United States from January to October 2025 based on Landsat 8 and Landsat 9 satellite data.** The missing pixels in the image are cloud masks.
